# Validated LC-MS/MS Method for the Determination of Scopoletin in Rat Plasma and Its Application to Pharmacokinetic Studies

**DOI:** 10.3390/molecules201018988

**Published:** 2015-10-19

**Authors:** Yingchun Zeng, Sha Li, Xiaohong Wang, Tao Gong, Xun Sun, Zhirong Zhang

**Affiliations:** 1 Key Laboratory of Drug Targeting and Drug Delivery Systems, Ministry of Education, West China School of Pharmacy, Sichuan University, Chengdu 610041, China; E-Mails: zych19900119@163.com (Y.Z.); 13980034382@163.com (S.L.); wangxiaohong0820@126.com (X.W.); gongtaoy@126.com (T.G.); xunsun22@gmail.com (X.S.); 2 West China School of Pharmacy, Sichuan University, No. 17, Block 3, Southern Renmin Road, Chengdu 610041, China

**Keywords:** scopoletin, LC-MS/MS, pharmacokinetics, rat plasma

## Abstract

A rapid, sensitive and selective liquid chromatography-electrospray ionization-tandem mass spectrometric method was developed and validated for the quantification of scopoletin in rat plasma. After the addition of the internal standard xanthotoxin, plasma samples were pretreated by a simple one-step protein precipitation with acetonitrile-methanol (2:1, *v/v*). Chromatographic separation was achieved on a Diamonsil ODS chromatography column using gradient elution with the mobile phase consisting of acetonitrile and 0.1% formic acid. The determination was performed by positive ion electrospray ionization in multiple reaction monitoring mode. The calibration curve was linear over the concentration range of 5–1000 ng/mL (*r* = 0.9996). The intra- and inter-day precision (RSD%) was less than 6.1%, and the accuracy (RE%) was from −3.0%–2.5%. This method was successfully applied to the pharmacokinetic research of scopoletin in rats after intravenous (5 mg/kg) or oral (5, 10 and 20 mg/kg) administration. The result showed that oral bioavailability with a dose of 5 mg/kg was 6.62% ± 1.72%, 10 mg/kg, 5.59% ± 1.16%, and 20 mg/kg, 5.65% ± 0.75%.

## 1. Introduction

**Figure 1:**
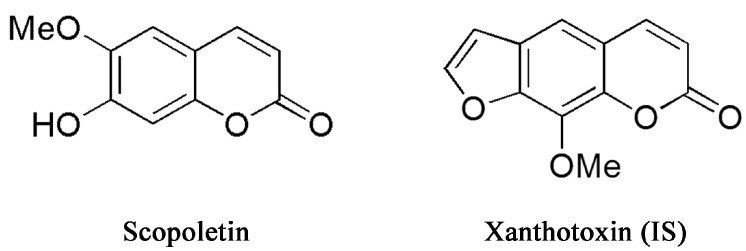
Chemical structures of scopoletin and xanthotoxin internal standard.

Caulis *Erycibes* (“*Dinggongteng*” in Chinese), a traditional Chinese medicine (TCM), is the dry rattan stem of *Erycibe obtusifolia* Benth or *Erycibe schmidtii* Craib. It is widely used to treat rheumatoid arthritis, hemiplegia, swelling and pain [1]. Scopoletin (6-methoxy-7-hydroxycoumarin; Figure 1) is an important pharmacologically-active constituent extracted from *Dinggongteng*. Numerous studies indicate that scopoletin exerts a variety of bioactive and pharmacological effects, such as anti-oxidative [2,3], anti-thyroid and anti-hyperglycemic [3], anti-hyperuricemic [4], anti-nociceptive [5] and anti-arthritic effects [6]. In addition, many studies have shown that scopoletin has anti-inflammatory effects [7,8,9,10]. The above research suggested that scopoletin could be an effective natural compound in further new drug investigation.

Recent publications have described several analytical methods for the determination of scopoletin in biologic samples using HPLC-UV [11] and LC-MS [12,13,14]. A few pharmacokinetic data of scopoletin after an oral administration of scopoletin (50 mg/kg) were acquired using HPLC-UV detection with the limit of quantitation as high as 165 ng/mL [11]. As this method has low sensitivity, the *in vivo* absorption characteristics of scopoletin could not be investigated in detail. Although an LC-MS method was developed for the determination of multiple components, including scopoletin in biosamples after oral administration of *Saussurea laniceps* extract [14], this LC-MS method also lacked sensitivity (50 ng/mL) and required a long elution time (>17 min). More importantly, other substances existing in the total herbal extract may affect the biological behavior of scopoletin because of the potential drug interaction [15], so this result could not reflect the absorptive properties itself. Furthermore, the pharmacokinetic behavior of scopoletin after intravenous administration has not been investigated. The pharmacokinetic studies of scopoletin were not conducted in other research in which the methods have several drawbacks, such as long elution time, narrow liner range and being unsuitable for plasma samples [12,13].

Therefore, the objective of the current study was to develop a selective, sensitive and rapid LC-MS/MS method for the quantification of scopoletin in rat plasma. Moreover, the method has been successfully applied to routine pharmacokinetic studies of scopoletin in rats after both oral (*p.o.*, per os) and intravenous (*i.v.*) administration. Meanwhile, oral absolute bioavailability of scopoletin was also studied for the first time.

## 2. Results and Discussion

### 2.1. Optimization of Mass Spectrometric and Chromatographic Conditions

**Figure 2:**
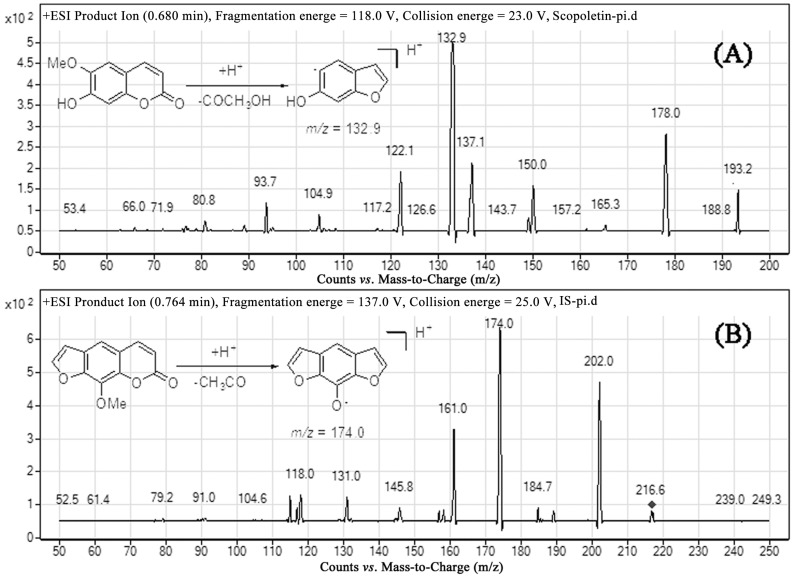
Fragment ion spectra of (**A**) scopoletin and (**B**) xanthotoxin.

Both negative and positive ion modes were tried, and positive ESI was found to be more sensitive for the determination of analytes. From the full scan mass spectra of scopoletin and xanthotoxin, the [M + H]^+^ ion with *m*/*z* 193.2 and 216.6, respectively, was selected as the precursor ion. Under the product ion scan mode, the most abundant fragments were recorded at *m*/*z* 132.9 for scopoletin and 174.0 for xanthotoxin, respectively (Figure 2). Therefore, quantitation was performed using multiple reaction monitoring (MRM) mode with target ions at *m*/*z* 193.2→132.9 for scopoletin and *m*/*z* 216.6→174.0 for xanthotoxin. Initially, we considered acetonitrile or methanol as the organic phase. Later, acetonitrile was chosen because it generated a shorter retention time and a better peak shape. However, this condition still led to peak tailing. To improve the peak shape, 1% formic acid was added to the aqueous phase. In order to achieve optimal separation in the probable shortest time, we developed a gradient elution program. Xanthotoxin was selected as the internal standard due to its similar extraction efficiency and ionization efficiency to scopoletin.

### 2.2. Optimization of Sample Preparation

An efficient sample preparation method is very important for accurate and reliable LC-MS/MS assays. A simple and rapid protein precipitation pretreatment was employed in our work. The mixed precipitant of acetonitrile-methanol (2:1, *v/v*) was chosen as the solvent for sample preparation, because it exhibited a better effect than acetonitrile and methanol alone. To sum up, peak shape, extraction recovery and other parameters met the requirements of EMA (European Medicines Agency) and the U.S. FDA (The Food and Drug Administration) [16,17].

### 2.3. Method Validation

#### 2.3.1. Selectivity

**Figure 3:**
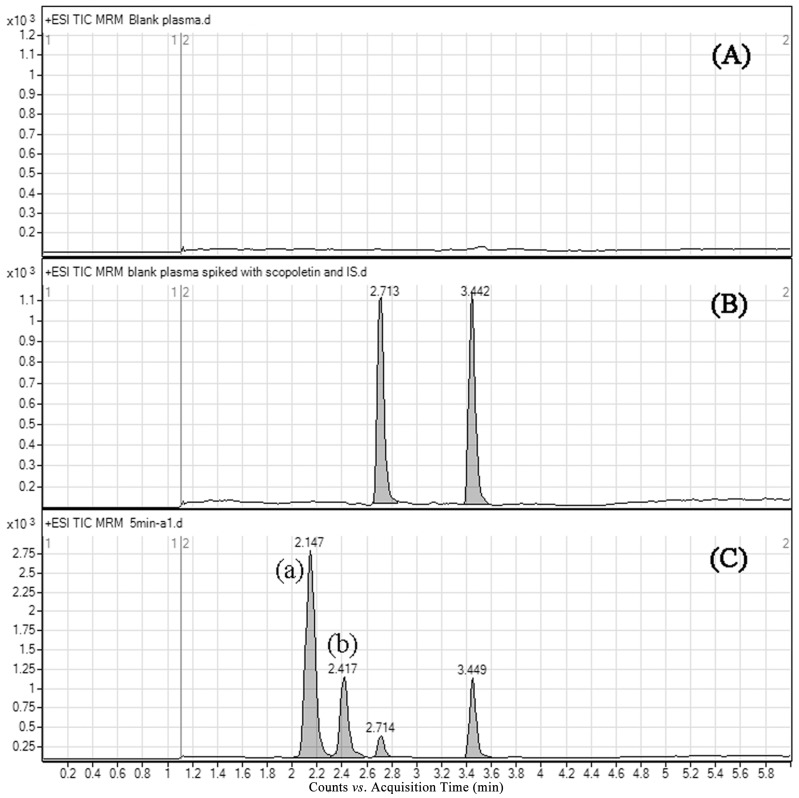
Representative multiple reaction monitoring (MRM) chromatograms of scopoletin and xanthotoxin in rat plasma: (**A**) a blank plasma sample; (**B**) a blank plasma spiked with 100 ng/mL of scopoletin and internal standard; (**C**) a plasma sample collected 5 min after oral administration of scopoletin; (**a**) scopoletin-glucuronide conjugate; (**b**) scopoletin-sulfate conjugate.

Typical chromatograms of blank rat plasma, blank sample spiked with scopoletin and xanthotoxin, and the plasma sample obtained after scopoletin administration to rats are shown in Figure 3. Scopoletin and IS were eluted at about 2.71 and 3.44 min, respectively. No endogenous interferences were observed for the retention times of the analytes.

In the present study, two metabolites were detected in rat plasma using MRM mode. Previous studies demonstrated that phenolic hydroxyl groups are easily affected by phase II metabolism [18]. Glucuronide ([M + H]^+^, *m*/*z* 369.1) and sulfate ([M + H]^+^, *m*/*z* 273.0) conjugates of scopoletin were detected in plasma samples using selected ion monitoring (SIM) mode under the developed chromatographic condition. Moreover, these conjugates had the same retention times with Metabolite (a) and Metabolite (b), respectively (Figure S1). According to the above results, we supposed that the metabolites were scopoletin-glucuronide Conjugate (a) and scopoletin-sulfate Conjugate (b) [14]. This phenomenon showed that the ester bonds of metabolites were broken to form the original drug after chromatographic separation under the current method. High temperature and pressure could provide a chance for the ester bond to break during atomization. Meanwhile, the analytes were completely separated from the metabolites and could be fully quantified (Figure 3).

#### 2.3.2. Linearity and Sensitivity

The calibration curves of scopoletin in rat plasma were obtained based on the peak area ratio of scopoletin to IS (*y*) *vs.* the scopoletin concentration (*x*), showing a good linear relationship over the range of 5–1000 ng/mL. A typical calibration curve equation was *y* = 0.01*x* − 0.0044 (*r* = 0.9996, *r* was the correlation coefficient). The lower limit of quantification (LLOQ) of scopoletin in rat plasma was 5 ng/mL; accuracy (RE%) was −7.6% and precision (RSD%) was 7.4%. The LOD, determined as a signal-to-noise ratio of 3:1, was found to be 1 ng/mL.

#### 2.3.3. Extraction Recovery and Matrix Effect

The absolute recovery and matrix effects of scopoletin and IS are shown in Table 1. The extraction recovery for scopoletin ranged from 93.9%–96.6% over the three QC concentration levels, while the extraction recovery of xanthotoxin was 92.3%. The matrix effect for scopoletin ranged from 101.8%–103.0%, and the matrix effect of xanthotoxin was 97.4%. These results suggested that the extraction method could provide high extraction efficiency, and there was no significant matrix effect on the method response to analytes.

**Table 1:** Recovery and matrix effect for scopoletin and IS in rat plasma (*n* = 5).

Compounds	Nominal Con. (ng/mL)	Recovery (Mean ± SD, %)	RSD (%)	Matrix Effect (Mean ± SD, %)	RSD (%)
Scopoletin	10	96.6 ± 1.7	1.8	101.8 ± 5.3	5.2
	100	93.9 ± 1.4	1.5	102.9 ± 3.2	3.1
	800	95.4 ± 0.7	0.7	103.0 ± 2.5	2.4
IS	100	92.3 ± 1.3	1.4	97.4 ± 4.7	4.8

Abbreviations: con., concentration.

#### 2.3.4. Precision and Accuracy

The accuracy and precision of the method were evaluated by determining replicate QC samples (10, 100 and 800 ng/mL) on three days. The intra-day and inter-day accuracy (RE%) ranged from −3.0%–2.5%, and the precision (RSD%) run varied between 3.2% and 6.1% (Table 2). The results demonstrated that this analytical method was precise and accurate.

**Table 2:** Precision and accuracy for scopoletin in rat plasma (*n* = 5).

Nominal Con. (ng/mL)	Intra-Day Run			Inter-Day Run		
	Measured Con. (Mean ± SD, ng/mL)	RSD (%)	RE (%)	Measured Con. (Mean ± SD, ng/mL)	RSD (%)	RE (%)
10	9.70 ± 0.51	5.3	−3.0	10.25 ± 0.62	6.1	2.5
100	100.48 ± 5.23	5.2	0.5	99.01 ± 3.68	3.7	−0.1
800	818.62 ± 38.31	4.7	2.3	817.64 ± 26.56	3.2	2.2

Abbreviations: con., concentration.

#### 2.3.5. Stability

The data for stability experiments are summarized in Table 3. These results suggested that scopoletin was rather stable under different storage conditions, and our LC-MS/MS method was applicable for routine analysis.

**Table 3:** Stability of scopoletin in rat plasma under different storage conditions (*n* = 5).

Storage Conditions	Nominal Con. (ng/mL)	Measured Con. (Mean ± SD, ng/mL)	RSD (%)	RE (%)
3 freeze-thaw cycles	10	10.36 ± 0.57	5.5	3.6
	100	101.66 ± 3.99	3.9	1.7
	800	804.58 ± 6.13	0.8	0.6
Short-term stability for 6 h (25 °C)	10	9.82 ± 0.49	5.0	−1.8
	100	101.39 ± 5.27	5.2	1.4
	800	812.33 ± 30.27	3.7	1.5
Long-term stability for 1 month (−20 °C)	10	9.30 ± 0.45	4.8	−7.0
	100	98.20 ± 3.59	3.7	−1.8
	800	813.78 ± 21.83	2.7	1.7
Long-term stability for 2 weeks (4 °C)	10	9.80 ± 0.43	4.4	−2.0
	100	99.84 ± 2.97	3.0	−0.2
	800	812.69 ± 21.48	2.6	1.6
Autosampler for 24 h (25 °C)	10	9.83 ± 0.55	5.6	−1.7
	100	97.62 ± 4.40	4.5	−2.4
	800	805.89 ± 24.31	3.0	0.7

Abbreviations: con., concentration.

#### 2.3.6. Dilution Integrity

Dilution integrity experiments were performed in five replicates by a four-fold dilution with blank plasma. The accuracy was 2.9%, and the precision was 2.4%. The result revealed that unknown samples with a higher concentration could be appropriately diluted with blank plasma and let the plasma concentration fall in the range of the standard curve for later analysis.

#### 2.3.7. Incurred Sample Reanalysis

About 90% (>67%) of the repeated sample results were within 20%. The results were acceptable, suggesting that this validated method was reproducible.

### 2.4. Pharmacokinetic Study and Oral Bioavailability

The optimized method was successfully applied to quantify scopoletin in rats following intravenous (5 mg/kg; *n* = 5) or oral administration (5, 10 and 20 mg/kg; *n* = 5 for each dose).

**Figure 4:**
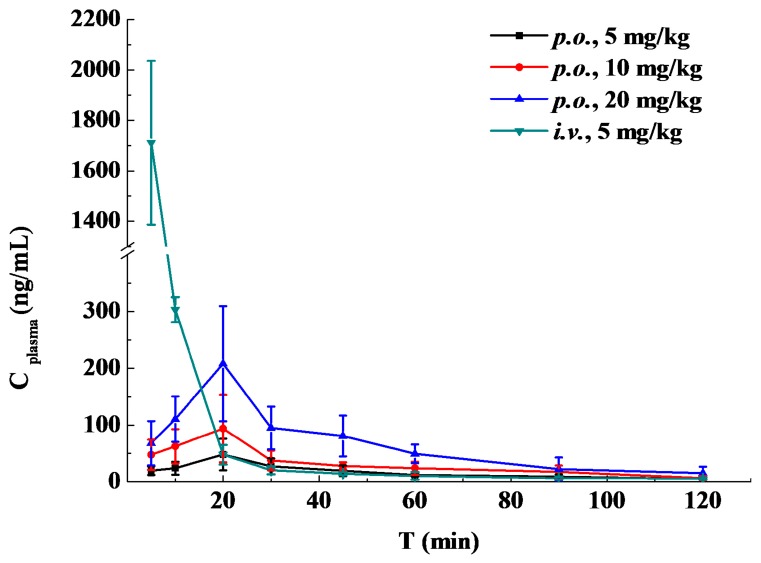
Mean scopoletin plasma concentrations over time after oral (*p.o.*, 5, 10 and 20 mg/kg) or intravenous (*i.v*., 5 mg/kg) administration in rats (*n* = 5).

The results of scopoletin concentration changing over time in rat plasma are summarized in Table 4, and the concentration-time curves are presented in Figure 4. The pharmacokinetic parameters were calculated by DAS 3.0 software. The main parameters for scopoletin in each group are given in Table 5. The statistical analysis showed that there was no significant difference in mean residence time (MRT), t_1/2_, T_max_ and clearance (CL) between the three *p.o.* groups (*p* > 0.05). A fast absorption process was found after oral administration of scopoletin, and it reached its peak concentration at around 20 min. The t_1/2_ after oral and intravenous administration suggested that scopoletin was eliminated quickly *in vivo*.

**Table 4:** Mean plasma concentrations of scopoletin (C_plasma_, ng/mL) over time (*n* = 5, mean ± SD).

*t* (h)	*i.v.* Administration	Oral Administration of the Indicated Dose		
	(5 mg/kg)	(5 mg/kg)	(10 mg/kg)	(20 mg/kg)
0.083	1711.13 ± 324.79	19.90 ± 7.70	47.80 ± 26.43	68.04 ± 38.88
0.167	303.00 ± 22.07	24.09 ± 11.36	62.61 ± 29.96	110.45 ± 40.16
0.33	47.98 ± 17.30	48.22 ± 27.74	93.85 ± 59.41	207.81 ± 101.26
0.5	20.58 ± 6.91	27.46 ± 14.06	37.95 ± 17.17	95.00 ± 37.45
0.75	14.45 ± 2.60	19.63 ± 9.48	28.09 ± 6.46	80.71 ± 35.88
1	10.67 ± 6.68	12.30 ± 2.94	24.22 ± 11.16	49.45 ± 16.46
1.5	6.31 ± 2.42	9.23 ± 3.98	17.68 ± 11.08	22.58 ± 20.41
2	5.79 ± 1.97	5.01 ± 1.38	6.86 ± 3.52	15.54 ± 11.34

**Table 5:** Pharmacokinetic parameters of scopoletin in rats (*n* = 5, mean ± SD). MRT, mean residence time; CL, clearance.

Parameter	*i.v.* Administration	Oral Administration of the Indicated Dose		
	(5 mg/kg)	(5 mg/kg)	(10 mg/kg)	(20 mg/kg)
AUC_0-t_ (μg /L·h)	617.780 ± 188.945	33.819 ± 6.640	61.984 ± 9.406	127.770 ± 16.874
AUC_0-∞_ (μg /L·h)	624.855 ± 184.496	41.363 ± 12.048	69.910 ± 16.189	141.112 ± 20.857
MRT_0-t_ (h)	0.066 ± 0.028	0.719 ± 0.102	0.685 ± 0.166	0.640 ± 0.164
MRT_0-∞_ (h)	0.116 ± 0.090	1.263 ± 0.700	0.948 ± 0.334	0.835 ± 0.299
t_1/2_ (h)	0.814 ± 0.326	0.935 ± 0.781	0.656 ± 0.379	0.539 ± 0.146
T_max_ (h)	NA	0.398 ± 0.093	0.331 ± 0.118	0.414 ± 0.188
CL (L/h/kg)	8.661 ± 2.829	128.781 ± 34.852	148.364 ± 29.053	144.164 ± 20.623
C_max_ (μg/L)	NA	49.786 ± 27.379	101.314 ± 52.201	217.324 ± 87.299

Abbreviations: NA, not applicable.

The oral bioavailability of scopoletin in rats was 6.62% ± 1.72% for 5 mg/kg, 5.59% ± 1.16% for 10 mg/kg and 5.65% ± 0.75% for 20 mg/kg, which was similar to the bioavailability of coumarin (3.40% ± 2.60%) [19]. Student’s *t*-test showed that oral bioavailability was statistically similar between the three *p.o.* groups. Previous studies showed that coumarin could be rapidly absorbed from the human gastrointestinal tract and extensively metabolized by the liver, so that only 2%–6% of coumarin reached the systemic circulation intact [19,20]. As a kind of coumarin analogue, the rapid absorption, metabolism and excretion of scopoletin in the body might be the reasons for the poor bioavailability.

## 3. Experimental Section

### 3.1. Chemicals and Reagents

Scopoletin (purity >98.0%) and xanthotoxin (purity >98.0%) as the internal standard (IS) were purchased from Chengdu Jin Zhe Bio-tech Co., Ltd. (Chengdu, China). HPLC-grade acetonitrile and methanol were supplied by Sigma-Aldrich (St. Louis, MO, USA). HPLC-grade formic acid was obtained from Tedia (Cincinnati, OH, USA). Ultra-pure water was prepared with a Milli-Q system (Millipore, Bedford, MA, USA).

### 3.2. Instrumentation and Analytical Conditions

The HPLC system consisted of a rapid resolution liquid chromatography system (1200 series, Agilent Technologies, Santa Clara, CA, USA) equipped with an SL binary pump, SL autosampler and degasser. The chromatographic separation was performed on a Diamonsil ODS column (100 mm × 4.6 mm, 3 μm) protected by a guard column (ODS, 5 μm). The mobile phase consisted of (A) 0.1% (*v*/*v*) formic acid solution and (B) acetonitrile. The optimal gradient elution was programmed as follows: 0.0–2.5 min, 10.0%→85% B; 2.5–3.0 min, 85.0%→10.0% B; 3.0–6.0 min, 10.0% B. The flow rate was 0.4 mL/min, and the column temperature was maintained at 30 °C.

An Agilent 6140 triple-quadrupole mass spectrometer equipped with an electrospray ionization (ESI) interface was used for mass spectrometric analysis. All quantifications were carried out in the positive ionization mode using multiple reaction monitoring (MRM). The MRM transitions selected for determination were *m*/*z* 193.2→132.9 for scopoletin and *m*/*z* 216.6→174.0 for xanthotoxin, respectively. The optimized fragmentation energy and collision energy were 118.0, 23.0 eV for scopoletin and 137.0, 25.0 eV for xanthotoxin, respectively. Nitrogen was used as the collision gas at 350 °C at a flow rate of 10 L/min. The nebulizer pressure was 30 psi, and the spray voltage was 4000 V. The dwell time was set at 300 ms.

### 3.3. Preparation of Stock and Working Solutions, Calibration Standards and Quality Control Samples

Stock solutions of scopoletin and IS were prepared in acetonitrile at concentrations of 1.0 mg/mL. A series of working standard solutions of scopoletin (0.05, 0.1, 0.2, 0.5, 1, 2, 5, 10 μg/mL) were prepared by diluting the stock solution with acetonitrile. Xanthotoxin working solutions were prepared in a similar way at concentrations ranging from 500–1000 ng/mL [21]. All stock solutions and working standard solutions were stored at 4 °C before use.

Calibration standards for scopoletin were prepared by spiking 10 μL of scopoletin working solution and 10 μL of IS working solution (1000 ng/mL) into 100 μL of blank plasma to yield final concentrations of 5, 10, 20, 50, 100, 200, 500 and 1000 ng/mL. Quality control (QC) samples were similarly prepared at concentrations of 10, 100 and 800 ng/mL for the low, medium and high level, respectively.

### 3.4. Sample Preparation

After thawing at room temperature, a 100-μL aliquot of plasma was spiked with 20 μL of IS working solution (500 ng/mL). Then, 300 μL of acetonitrile-methanol (2:1, *v*/*v*) were added, and the mixture was vortexed for 3 min. The well-vortexed solutions were then centrifuged at 15,500× *g* for 10 min, and the supernatants were filtered through a 0.22-μm hydrophobic membrane (Tianjin Heng’ao Technological Industry Group Ltd., Tianjin, China). Finally, 3 μL of the sample were injected into the LC-MS/MS system for analysis.

### 3.5. Method Validation

The LC-MS/MS method was validated according to the guidelines of EMA and the U.S. FDA for selectivity, linearity, sensitivity, recovery, matrix effect, accuracy, precision, stability, dilution integrity and incurred sample reanalysis (ISR) [16,17].

#### 3.5.1. Selectivity

Blank plasma samples collected from six different rats, plasma samples spiked with scopoletin and IS and plasma samples after gavages of scopoletin were tested to investigate the potential interferences in the chromatography peak region for analytes.

#### 3.5.2. Linearity and Sensitivity

Calibration curves were constructed by assaying calibration standards spiked with eight nominal scopoletin concentrations (5, 10, 20, 50, 100, 200, 500 and 1000 ng/mL) on three separate days. The peak-area ratio of scopoletin to IS was plotted *vs.* scopoletin concentration; the linear regression equation and correlation coefficient were calculated by weighted (1/*x*^2^) least-squares linear regression analysis. The lower limit of quantification (LLOQ) was defined as the lowest concentration of the calibration curve at which the accuracy did not deviate by more than 20%, and the signal-to-noise ratio (S/N) was at least 10:1. The limit of determination (LOD) was determined as the lowest concentration in plasma with a signal-to-noise ratio of 3:1.

#### 3.5.3. Extraction Recovery and Matrix Effect

The extraction recovery and matrix effect of scopoletin were assessed by assaying three sets of samples at three concentration levels (10, 100 and 800 ng/mL). Scopoletin and IS were: (A) spiked into plasma before extraction; (B) spiked into plasma after extraction; or (C) spiked directly into equivalent volumes of acetonitrile-methanol (2:1, *v*/*v*). The extraction recovery, also known as absolute recovery, was determined by comparing the scopoletin peak area obtained from Set A samples with that from Set C samples. The matrix effect was evaluated by comparing the peak area of Set B samples to that of Set C samples.

#### 3.5.4. Precision and Accuracy

In the current study, the intra-day and inter-day precision and accuracy were evaluated by analyzing five replicates of the QC samples (10, 100 and 800 ng/mL) on three consecutive days. The accuracy and precision were expressed by the relative error (RE%) and relative standard deviation (RSD%), respectively.

#### 3.5.5. Stability

The stability of scopoletin in rat plasma was investigated by analyzing QC samples at concentrations of 10, 100 and 800 ng/mL after storage under different conditions: (1) the freeze-thaw stability of scopoletin in rat plasma was evaluated after three freeze-thaw cycles; (2) the short-term stability was determined by placing plasma samples at room temperature (25 °C) for 6 h; (3) the long-term stability was tested after storing the samples at −20 °C for 1 month or at 4 °C for 15 days; (4) the post-preparative stability was evaluated after keeping the samples in the autosampler at 25 °C for 24 h.

#### 3.5.6. Dilution Integrity

The range of the standard curve was defined, and the estimation of the concentration in unknown samples by extrapolation of the standard curve was not recommended. Samples above the highest standard concentration (above ULOQ, upper limit of quantification) could be diluted with the same blank biological matrix [16,17]. The dilution integrity experiment was carried out by analyzing five replicates of plasma samples (2000 ng/mL). Samples were diluted 4 times with blank rat plasma and mixed evenly, and then, 100 μL of plasma were transferred and dealt with as described for the sample preparation. According to the dilution factor (×4), we could calculate the initial plasma drug concentration. The accuracy and precision of these samples must be within ±15% [16].

#### 3.5.7. Incurred Sample Reanalysis

ISR assessment samples were chosen in the vicinity of t_max_ (0.33 h) and the elimination phase (2 h). All rat samples of *p.o.* groups at the above time points were reanalyzed. The percentage difference was calculated using the mean of the original and repeat results as follows: difference (%) = (repeat − original)/mean × 100%. Two-thirds (67%) of the repeated sample results should be within 20%.

### 3.6. Application to Pharmacokinetic Study

The study was performed in accordance to the Guidelines for animal Experiments of Sichuan University (Chengdu, Sichuan, China). Male Sprague-Dawley rats (200 ± 10 g) were obtained from the Experimental Animal Center of West China, Sichuan University. Animals were housed in an environmentally-controlled breeding room (temperature: 23–26 °C; relative humidity: 40%–60%; noise: <60 dB; 12 h dark-light cycle) for at least 1 week before experimentation to adapt to housing conditions. Rats were fasted for 12 h prior to the experiments, while water was always available.

The rats were randomly divided into four groups to diminish the individual variation. The *i.v.* group was given scopoletin intravenously at a dose of 5 mg/kg, while the three *p.o.* groups were administered orally at a dose of 5 mg/kg (low dose), 10 mg/kg (medium dose) and 20 mg/kg (high dose), respectively. DMSO-PEG400-ethanol-5% aqueous glucose (3:3:2:2, *v*/*v*/*v*/*v*) and 0.5% carboxymethyl cellulose sodium (CMC-Na) were used as solvents to administer scopoletin in the *i.v.* group and *p.o.* groups, respectively.

Blood samples (300 μL) were collected from the suborbital vein into 0.5-mL heparinized polythene tubes at 5, 10, 20, 30, 45, 60, 90, 120 min after scopoletin administration. All blood samples were immediately centrifuged at 2000× *g* for 5 min. One hundred microliters of plasma supernatant were transferred into 1.5-mL polythene tubes and stored at −20 °C until analysis.

The pharmacokinetic parameters were calculated by Drug and Statistics Software (DAS 3.0; Mathematical Pharmacology Professional Committee of China, Shanghai, China). The main parameters included the area under the plasma concentration time curve during the period of observation (AUC_0–*t*_), the area under the plasma concentration time curve from zero to infinity (AUC_0–∞_), the mean residence time (MRT), half-life (t_1/2_) and clearance (CL). Oral bioavailability was determined by comparing AUC_0–∞_ of scopoletin after oral administration to that after intravenous administration. It was calculated as follows: *F* = (AUC*_p.o._* × Dose*_i.v._*)/(AUC*_i.v._* × Dose*_p.o._*) × 100%.

## 4. Conclusions

A simple, sensitive and selective LC-MS/MS method was developed and validated for the quantification of scopoletin in rat plasma. This method was proven to be linear over the concentration range of 5–1000 ng/mL. The total analysis time of scopoletin and IS was 6 min, which significantly improved the analysis efficiency. The validated method was successfully applied to the pharmacokinetic study of scopoletin in rats after both oral and intravenous administration for the first time. The pharmacokinetic and oral bioavailability studies of scopoletin will provide helpful information for new drug developments and applications.

## Supplementary Materials

Supplementary materials can be accessed at: http://www.mdpi.com/1420-3049/20/10/18988/s1.
