# Supplementary material for: Validated LC-MS/MS Method for the Determination of Scopoletin in Rat Plasma and Its Application to Pharmacokinetic Studies
Source: Molecules. 2015 Oct 19;20(10):18988–9001. doi: 10.3390/molecules201018988 (PMC6332412; doi:10.3390/molecules201018988)
Supplement: Supplementary file 1 [file molecules-20-18988-s001.pdf]

Supplementary Materials

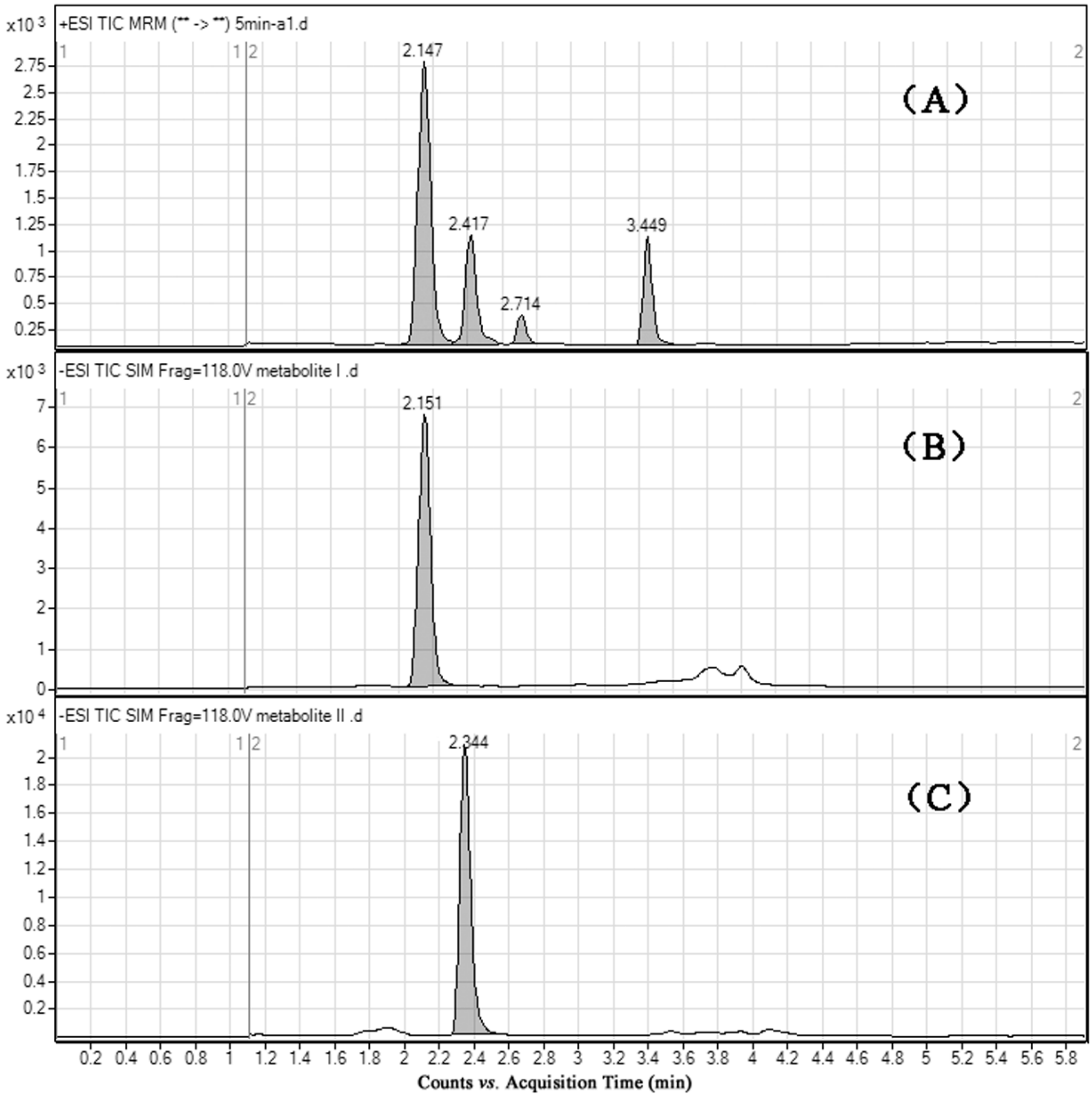

Figure S1. Representative chromatograms of scopoletin/metabolites in rat plasma: (**A**) MRM chromatogram of scopoletin and IS; (**B**) SIM chromatogram of scopoletin-glucuronide conjugate; (**C**) SIM chromatogram of scopoletin-sulfate conjugate.
